# Crisis of objectivity: using a personalized network model to understand maladaptive sensemaking in a patient with psychotic, affective, and obsessive-compulsive symptoms

**DOI:** 10.3389/fpsyg.2024.1383717

**Published:** 2024-08-06

**Authors:** Aleš Oblak, Matic Kuclar, Katja Horvat Golob, Alina Holnthaner, Urška Battelino, Borut Škodlar, Jurij Bon

**Affiliations:** ^1^Laboratory for Cognitive Neuroscience and Psychopathology, University Psychiatric Clinic Ljubljana, Ljubljana, Slovenia; ^2^Department of Psychiatry, Faculty of Medicine, University of Ljubljana, Ljubljana, Slovenia; ^3^Faculty of Slovenian and International Studies, New University, Ljubljana, Slovenia

**Keywords:** schizoaffective disorder, personalized network model, enactive psychiatry, psychiatric comorbidity, obsessive compulsive disorder, qualitative phenomenology, RDoC

## Abstract

**Introduction:**

Psychiatric comorbidities have proven a consistent challenge. Recent approaches emphasize the need to move away from categorical descriptions of symptom clusters towards a dimensional view of mental disorders. From the perspective of phenomenological psychopathology, this shift is not enough, as a more detailed understanding of patients’ lived experience is necessary as well. One phenomenology-informed approach suggests that we can better understand the nature of psychiatric disorders through personalized network models, a comprehensive description of a person’s lifeworld in the form of salient nodes and the relationships between them. We present a detailed case study of a patient with multiple comorbidities, maladaptive coping mechanisms, and adverse childhood experiences.

**Methods:**

The case was followed for a period of two years, during which we collected multiple streams of data, ranging from phenomenological interviews, neuropsychological assessments, language analysis, and semi-structured interviews (Examination of Anomalous Self Experience and Examination of Anomalous World Experience). We analytically constructed a personalized network model of his lifeworld.

**Results:**

We identified an experiential category “the crisis of objectivity” as the core psychopathological theme of his lifeworld. It refers to his persistent mistrust towards any information that he obtains that he appraises as originating in his subjectivity. We can developmentally trace the crisis of objectivity to his adverse childhood experience, as well as him experiencing a psychotic episode in earnest. He developed various maladaptive coping mechanisms in order to compensate for his psychotic symptoms. Interestingly, we found correspondence between his subjective reports and other sources of data.

**Discussion:**

Hernan exhibits difficulties in multiple Research Domain Criteria constructs. While we can say that social sensorimotor, positive valence, and negative valence systems dysfunctions are likely associated with primary deficit (originating in his adverse childhood experience), his cognitive symptoms may be tied to his maladaptive coping mechanisms (although, they might be related to his primary disorder as well).

## 1 Introduction

A young man enters a fast-food restaurant. The waitress recognizes him as a regular, but he nonetheless avoids her, stepping aside, pretending to be interested in the packets of mustard. He is buying time, preparing for the social interaction. When he orders his sandwich, he does not look at the waitress’ face. He feels terrified, self-conscious, embarrassed. He knows he is wrong. He knows he is a child of a mother with schizophrenia. He experienced a psychotic episode himself. He is haunted by the memories of monsters that prey on at night, of the voices commanding him to peel off his skin, of his mirror image that took on a life of its own and started to mock him. The interaction with the waitress is pleasant enough. Still, he gives her an unreasonably large tip as an apology because she had to endure his wrongness. The knowledge of his wrongness makes him unable to cross a road as he is not sure that he will not walk into traffic, or ascend a staircase knowing that he will not suddenly decide to simply throw himself down the stairs. To alleviate the wrongness, he drinks, he smokes weed.

Psychiatric disorders are commonly associated with a heterogenous clinical presentation, such as the one described above, which makes both treatment and research challenging (Allsopp et al., 2019). Two main classifications of mental health disorders, the Diagnostic and Statistical Manual of Psychiatric Disorders (DSM) and International Classification of Diseases (ICD), are criticized for classifying disorders based on clinical descriptions as there is overlap among the symptoms and biological features of disorders (Lilienfeld and Treadway, 2016). Different pathophysiological mechanisms may therefore lead to the same diagnosis (Cuthbert and Kozak, 2013; Sanislow, 2016). Descriptive classification of psychiatric disorders also fails to consider heterogeneity within each condition for different persons and time course (Wardenaar and De Jonge, 2013; Feczko et al., 2019). The problem of disorder classification partially contributes to the phenomenon of psychiatric comorbidity. As many as 45 % of patients satisfy the criteria for more than one disorder in a year (Allsopp et al., 2019). In fact, diagnostic systems DSM-III to DSM-5 have been criticized for simplifying diagnostic categories, which can result in overlooking a correct diagnosis or falsely diagnosing multiple comorbidities. One of the general criticisms of DSM categorization system that we find in the phenomenological literature is also that it treats the various symptoms as disparate parts rather than an unified whole (Stanghellini and Mancini, 2017).

Nordgaard et al. (2023) provide a conceptual analysis of psychiatric comorbidity. In somatic medicine, comorbidity refers to the co-occurrence of two distinct disease entities, each of which having a known etiology or pathology (Kaplan and Feinstein, 1974). However, in psychiatry, etiology is typically unknown and symptoms that are unique to only one disorder are rare. Nordgaard et al. (2023) further point out that - specifically in research - the notion of psychiatric comorbidity assumes independence of disease entities. What is commonly omitted is the idea of diagnostic hierarchy (i.e., the idea that certain diagnoses should not be made in the presence of other specific disease entities; Pincus et al., 2004; Ghaemi, 2018; Kotov et al., 2018). Nordgaard et al. (2023) acknowledge that true comorbidity may not even exist in psychiatry, while diagnosing comorbidities is associated with several problems, such as polypharmacy (Pjevac and Korošec Hudnik, 2023; Korošec Hudnik et al., 2024), higher risk of misdiagnosis, and misinterpretation of empirical findings in research where inclusion criteria are too liberal.

Several methodological frameworks have been proposed in order to tackle the problem of symptom heterogeneity. The Research Domain Criteria (RDoC) is an investigative framework that proposes to identify endophenotypes mediating psychiatric disorders (Insel and Cuthbert, 2009). Endophenotype refers to the intermediate level of description between genetics, and the behavioral and phenomenological signs of psychiatric disorders (Cuthbert and Kozak, 2013). RDoC claims that psychiatric disorders are deviations of otherwise normal dimensions of human psychological functioning. These dimensions include cognition, positive affect, negative affect, physiological arousal, and social psychology (Cuncic, 2020). RDoC subscribes to explanatory pluralism, that is considering different levels of description (e.g., genetic, electrophysiological, behavioral, phenomenological) as different insights into the same process rather than one of them being epistemologically superordinate (Cuthbert and Insel, 2013). The transition towards dimensional models, however, has been criticized from the perspective of phenomenological psychopathology, citing poor conceptual clarification of psychiatric disease entities (Parnas, 2014).

Recently, one approach towards a more comprehensive account of psychiatric disorders was put forth by De Haan (2020). She argues that the central property of psychiatric disorders are maladaptive patterns of sensemaking. De Haan (2020) proposes that methodologically, we might be able to address sensemaking in psychiatric disorders by constructing personalized network models (PNM). PNMs are depictions of different aspects of a patient’s functioning that influence and modulate each other, leading to changes in psychiatric symptoms. PNMs represents phenomena in terms of nodes (a relevant variable) and edges (connections between them). De Haan (2020) outlines four domains that are to be included in a PNM: biological, social, experiential, and existential (i.e., a person’s attitude towards their broader situation).

So far, only one empirical study demonstrated the use of PNM. Larsen et al. (2022a,b) used it to investigate the relationship between psychosis and cannabis use in a longitudinal dual case study. They conducted six interviews per patient. Analytically, they used the collected qualitative material to construct a web of relationships between salient aspects of the patients’ lives, in particular in relation to their cannabis use. For one patient, stopping cannabis use proved challenging due to his proximity to the drug (e.g., frequent use by his partner, engagement in the local crime scene). For the second patient, the cessation of cannabis was related to an intricate feedback loop wherein desensitization (which initially proved to be a useful coping mechanism) made her environment less salient. Further, she was concerned with obesity which was maintained through increased appetite under the acute effect of cannabis.

However, De Boer et al. (2022) have criticized network models, arguing that they suffer from a boundary problem; that is, the question of what domains to include in the network analysis of a given patient, as well as how can we even differentiate different domains from one another? One of their arguments is that network analysis favors perspectivism: epistemological position that claims that the construction of scientific bodies of knowledge must take into account the researchers’ perspectives as well. A patient may feel that the core of their problems is, for example, them struggling in school. Taking this belief seriously, by necessity constrains our potential interventions, and as such we may miss out on the optimal change for that specific patient. The second major critique of network analysis refers to the relevancy of the patterns emerging from it. Is the overall knowledge about the psychopathology constructed by a network analysis relevant for the patient, the clinician or the broader psychiatric community?

To recapitulate: Not only is the phenomenon of psychiatric comorbidity relevant and difficult to tackle, its proposed solutions are fraught with problems as well. The present paper attempts to contribute to these discussions by refining the PNM approach using contemporary methods in qualitative phenomenology. A case report is presented. The case was chosen for two reasons: a) the patient presents with several symptom constellations and b) was interested in participating in a longitudinal study. The present paper has three main research goals:

(1) It presents a proof of principle of how PNMs could be integrated into qualitative phenomenological methodology;
(2) It evaluates whether using novel frameworks in psychopathology (RDoC and PNMs) can assist us in psychiatric diagnosis;
(3) It presents a novel phenomenological category (what we term crisis of objectivity) that was identified with PNMs.

Due to the complexity of developing novel methodological frameworks, we opted for demonstrating our understanding of PNMs (as originally developed by De Haan, 2020) with a case study. In doing so, we are following similar approaches in phenomenological psychopathology wherein data from single, highly engaged patients are used to resolve technical or conceptual issues (de Haan and Fuchs, 2010; Henriksen et al., 2010; Stanghellini and Rosfort, 2013; Luhrmann and Marrow, 2016; Wigand et al., 2018; Englebert et al., 2019). Thus, rather than focusing on the clinical relevance of the case itself, we wanted to use the data from this patient to demonstrate how the conceptual framework of PNMs can be integrated with modern methods in qualitative research and phenomenological psychopathology to better understand psychiatric comorbidity.

## 2 The case

The patient was recruited from an ongoing project of testing the efficacy of online psychotherapy. Thus, contact with him was conducted over video conference. The patient, who we will anonymize as Hernan, is a 27-year-old man of Western European origin living abroad. Formally trained as a journalist, he has recently left his journalistic job to pursue a career as an online content creator. He lives with his husband. Hernan reports a life-long history of psychosis-like experiences, ranging from auditory and visual hallucinations and common periods of dissociation. He frequently experiences nighttime hallucinations and parasomnias during which he talks, screams, and attacks others. His father was imprisoned for drug-related crimes. Hernan believes that his mother suffers from schizophrenia and therefore questions his own perception of reality. Because of imperative auditory hallucinations, he often scratched his skin to the point of injury. He experiences frequent obsessive compulsive symptoms (OCS). These consist of him continuously checking his environment in potentially dangerous situations.

At seventeen, Hernan was diagnosed with schizoaffective disorder and prescribed aripiprazole, which he declined taking. He has not received any psychiatric treatment later on, citing high costs of medical services as the main reason. In the past, he attended psychotherapy sessions for OCS, which ended after a few seasons for the same reason. Prior to being included in this study, he has again started with psychoanalytic psychotherapy. Between the ages of ten and fifteen, he attempted suicide several times and had occasional outbursts of anger and heteroagressive thoughts. Hernan still has suicidal thoughts and egodystonic intrusive thoughts about buying a gun, but does not intend to commit suicide. He agreed to suicide prevention contract during the therapy.

He remembers being a sad, quiet, angry and lonely child who was discouraged from showing emotions by his mother. Together with his brothers she often ridiculed him. His mother could not accept him being gay, so Hernan pretended to be confused about his sexuality to avoid conflicts. When he broached the subject again at nineteen, his family reacted with aggression. Following a domestic altercation, Hernan left in a hurry and was homeless for nine months.

After turning 18, Hernan smoked marijuana multiple times a day and has taken methylenedioxymethamphetamine (MDMA) and lysergic acid diethylamide (LSD)many times. At present he regularly takes delta-8-tetrahydrocannabinol (delta-8 THC) (200 mg/2 weeks). His psychotic symptoms precede the beginning of the use of recreational drugs. He admits to drinking alcohol excessively during social events in the past, but denies regular use. In the past five years he abstains from alcohol. This has been confirmed by his partner in a conversation with the principal investigator.

## 3 Materials and Methods

Qualitative material was collected using in-depth phenomenological interviews derived from multiple methodological frameworks, predominantly micro-phenomenology (Petitmengin, 2006), interpretative phenomenological analysis (Smith et al., 2022), and constructivist grounded theory (Charmaz, 2014). Hernan’s symptoms were additionally assessed using the Examination of Anomalous World Experience (EAWE; Sass et al., 2017) and Examination of Anomalous Self Experience (EASE, Parnas et al., 2005) semi-structured interviews. EASE and EAWE items were scored as 0 (absent or questionably present) or 1 (definitely present, covering all severity levels).

The interviews followed a funnel-shaped structure, wherein we started with a general discussion on a specific aspect of Hernan’s life. After identifying specific aspects of experience (e.g., a symptom), we followed the guidelines of micro-phenomenological interview to examine the episodes in detail. We additionally collected descriptions of Hernan’s baseline experiences in more stressful periods of his life (e.g., when he spent nine months being homeless).

In total, 19 interviews were conducted with Hernan over an 18-month period. Two of the sessions were dedicated to the EAWE and EASE interviews. 16 interviews (including one session for the EAWE interview and one for a debriefing of the project) were conducted by a researcher with several years of experience in conducting phenomenological interviews (AO). EASE was conducted by a clinician trained in this method (AH). The medical history was taken by a psychiatry resident (KHG) in two interview sessions. Throughout the duration of the study, Hernan received supportive psychotherapy from a licensed psychotherapist (MK).

Following the RDoC approach, Hernan was evaluated on multiple psychological domains, using the adjusted version of the miniRDoC battery (first presented in Förstner et al., 2022), consisting of questionnaires and a cognitive task. Positive affect was evaluated using the *drive*, *fun-seeking* and *reward responsiveness* subscales of the Behavioral Inhibition System and Behavioral Approach System scale (BIS/BAS; Carver and White, 1994), and the *positive subscale* of the Positive and Negative Affect Schedule (PANAS; Crawford and Henry, 2004). Negative affect was evaluated using the *behavioral inhibition system* subscale of BIS/BAS, the *negative* subscale of PANAS, and the *phobic anxiety* subscale of the Symptom Checklist (SCL-90; Maruish, 2000). His social cognition was evaluated using the *getting along* and *participation* subscales of the World Health Organization Disability Assessment Schedule (WHODAS 2.0; Ustun et al., 2010), and *interpersonal sensibility* and *anger/hostility* subscales of the SCL-90. His sensorimotor cognition was evaluated using the *somatization* subscale of SCL and *mobility* subscale of WHODAS. His hot cognition was evaluated using the Emotion Regulation Questionnaire (ERQ; Gross and John, 2003) consisting of two dimensions: *expressive suppression* and *cognitive reappraisal*; the *cognition* subscale of WHODAS, and the Barratt Impulsiveness Scale (Barratt, 1965). Finally, cold cognition was evaluated using a verbal 2-back task. The 2-back task consisted of a letter appearing in the middle of the computer screen for 2.0 seconds. Hernan had to indicate, by button press, whether the letter was equal to or different from the letter that appeared on screen two trials previous. Performance accuracy (correct VS incorrect) was collected.

### 3.1 Analysis

The interviews were transcribed verbatim.^1^ The qualitative material was analyzed according to interpretative phenomenological analysis (Smith et al., 2022) in two phases. The first phase consisted of inductive-deductive coding of interview transcripts. In qualitative research, coding refers to the process of assigning more general, descriptive tags to sections of raw texts (Charmaz, 2014). For inductive-deductive approach we analyzed the text according to preexisting concepts from the (psychopathological, psychiatric) literature (e.g., *hallucination*), while at the same time, paying attention to the data that may question or re-examine existing concepts (e.g., *crisis of objectivity*) (Flick and Flick, 2011). A codebook was constructed in which all the relevant categories are described according to the following elements: (a) telling name; (b) relationship to other categories; (c) meaningful examples; and (d) potential additional comments (Nelson, 2017). The codebook is made available at: https://osf.io/dj8pt/.

In the second phase of analysis, we identified the relationships between different experiential categories in order to construct a PNM. PNM refers to a network of all the salient aspects of the patient’s life as well as the explanation of the connections between them. For the construction of the PNM, we started with the experiential categories yielded by inductive-deductive coding (forming the nodes of the PNM). Then, the interview transcripts were re-analyzed so as to identify the relationships between individual categories. Due to the novelty of the PNM approach, we adopted a simplifying assumption, wherein we searched for two types of relationships between categories: *upregulation* and *downregulation.* If category A upregulates category B, category B becomes more expressed. If category A downregulates category B, category B becomes less expressed. We assumed a rhizomatic structure to Hernan’s PNM: All categories could, in principle, be connected to any other category. Further, the relationships between categories were assumed to be directed. Thus, category A could regulate category B, but category B could also regulate category A. Relationships between the categories had to be grounded in the data in order to be considered valid. Further, we only considered those relationships admissible that were well-grounded: that is, that occurred in multiple interview sessions. Relationships between categories that could only be grounded in a single experiential episode were discarded.

We divided Hernan’s PNM into five domains: sensemaking (i.e., the stance he takes towards his own existential situation), symptoms, developmental factors, biological factors, and social factors. An important caveat has to be made regarding these domains. Since the only source of data that was directly accessible to us was phenomenological, only relationships pertaining to the experiential level of description were explicated. For example, Hernan’s mother was diagnosed with schizophrenia. As such, there is likely a genetic component to his disorder. However, this relationship remained unspecified, as we only had access to how Hernan reflectively makes sense of his family background.

We additionally analyzed the transcripts using keyword analysis. We extracted openly available interview data from two qualitative phenomenological studies on the sense of realness (Oblak et al., 2021, 2022). In Oblak et al. (2021) the sense of realness was explored in the normative population. In Oblak et al. (2022), a transdiagnostic sample of “altered” experiences of realness was collected (ranging from mystical, psychedelic, to psychopathological experiences). The transcripts of Hernan’s interviews were searched for the root morphemes of five keywords that we commonly observed in his reports: *rationality, truth, objectivity, reality*, and *fact.* A visual inspection was performed to remove instances where these words were used by the interviewers. Each root morpheme was expressed as an average occurrence of the word per interview. To validate this keyword analysis, we performed a statistical analysis on the data derived from the two groups. The data were tested for parametric assumptions. For all keywords, Shapiro-Wilk’s test revealed that normality was violated. Mann-Whitney rank test was used to estimate the difference between the two groups. FDR correction for multiple comparisons was used. The data from the two groups were then merged (i.e., we obtained a transdiagnostic sample). The percentile rank for Hernan’s word use was then determined.

Hernan’s symptoms were evaluated using the Schizotypal Personality Questionnaire (SPQ-32; Davidson et al., 2016), consisting of the *ideas of reference, suspiciousness, no close friends, constricted affect, eccentric behavior, social anxiety, magical thinking, odd speech*, and *unusual perception* subscales; the Yale-Brown Obsessive Compulsiveness Scale (Y-BOCS; Storch et al., 2015), consisting of *obsessions* and *compulsions* subscales; the Patient Health Questionnaire (PHQ-9; Kroenke et al., 2001), examining depression, and General Anxiety Disorder (GAD-7; Swinson, 2006), examining anxiety, Rumination Response Scale (RRS; Nolen-Hoeksema, 2000), consisting of *brooding* (maladaptive self-related cognition) and *reflective pondering* (adaptive self-related cognition). SCL subscales for *OCD, depression, anxiety, psychoticism* and *paranoid ideation* were used. Childhood Trauma Screener (CTS; Glaesmer et al., 2013) was used to test for adverse childhood experience. Finally, the total score on WHODAS was used to evaluate the general degree of Hernan’s functional impairment. Norms were derived from the patients from our clinical practice. The exceptions are norms for ERQ, which were obtained from Barrios and Olalde-Mathieu (2021), Y-BOCS, which were obtained from Fink (2018), and SPQ-32, which were obtained from https://faculty.lsu.edu/asap/normative-data.php.

## 4 Results

### 4.1 Symptoms: clinical scales

Hernan’s symptoms are summarized in Table 1. Hernan’s Y-BOCS scores correspond to mild OCS. His PHQ score corresponds to the presence of moderate depressive symptoms. His GAD score corresponds to severe anxiety. Analyses of the factor structure of RRS suggest that rumination can be subdivided into *reflective pondering* (adaptive response), and *brooding* (maladaptive response). On reflective pondering, Hernan ranks in the 74th percentile, whereas on brooding, he ranks in the 90th percentile (based on the group of all participants in our lab who had completed RRS; *N* = 275).

**TABLE 1:** Hernan’s symptoms as assessed by Schizotypal Personality Questionnaire (SPQ-32), Yale-Brown Obsessive Compulsiveness Scale (Y-BOCS), Patient Health Questionnaire (PHQ-9), Generalized Anxiety Disorder Assessment (GAD-7), Symptom Checklist (SCL-90), WHO Disability Assessment Schedule (WHODAS 2.0), Rumination Response Scale (RRS), and Childhood Trauma Screener (CTS).

Clinical scale	Hernan’s score (percentile rank)
**SPQ-32**	
Ideas of reference	7 (16)
Suspiciousness	9 (38)
No close friends	12 (93)
Constricted affect	11 (87)
Eccentric behavior	16 (99)
Social anxiety	4 (20)
Magical thinking	0 (16)
Odd speech	9 (55)
Unusual perception	12 (100)
**YBOCS**	
YBOCS (Obsessions)	15 (NA)
YBOCS2 (Compulsions)	14 (NA)
PHQ-7	12 (66)
GAD-9	19 (74)
**RRS**	
Reflective pondering	11 (75)
Brooding	19 (90)
**SCL**	
OCD	15 (84)
Depression	26 (96.2)
Anxiety	20 (67.8)
Psychoticism	15 (97.8)
Paranoid ideation	4 (55.8)
CTS	112 (99)
WHODAS - Sum score	98 (91.6)

### 4.2 Research domain criteria perspective

Hernan exhibits scores in the middle range for sensorimotor, negative valence, and positive valence systems. Notably, he demonstrates high scores in behavioral approach but consistently low scores in social processes, reflecting feelings of alienation and isolation. Although he scores in the 99th percentile for anger/hostility (i.e., suggesting lack of these feelings), he may have presented socially desirable responses. The cognitive systems domain is ambiguous, with reasonably high performance on the 2-back task (83rd percentile), high expressive suppression, and moderately low impulsivity. However, he faces challenges in everyday cognitive functioning and employs cognitive reappraisal less frequently.

### 4.3 Hernan’s speech and conduct during interviews

In interviews, Hernan was polite but avoided eye contact, often clutching a pillow for comfort and occasionally scratching himself. Apart from one instance of dissociation, interviews took place without complications. However, Hernan often expressed a desire for researchers’ approval. Given his tendency for socially desirable answers in the early sessions, we placed less importance on those interviews in the analysis. Hernan has a divergent style of thinking with occasionally disorganized speech. He is noticeably preoccupied with his metacognition and sense of reality. We noticed that he commonly uses terms related to epistemology, which we confirmed through quantitative keyword analysis.

Table 2 summarizes how Hernan’s use of epistemological terms compares to participants in two qualitative phenomenological studies investigating the sense of realness. Firstly, we see that there is a significant difference in the use of terms relating to rationality, fact and objectivity between a group recruited from the normative population and participants who experience an altered sense of presence. Hernan is in the 98th percentile for the use of the keyword rationality, in the 93rd percentile for the keyword fact, and 100th percentile for the keyword objectivity

**TABLE 2:** Comparison of epistemological keywords between Hernan, participants from the normative population, and participants who had experienced an altered sense of realness.

Keyword	Hernan [N/interview, percentile rank]	Normative experience (*N* = 30) [mean, SD] (Oblak et al., 2021)	Altered experience (*N* = 14) [mean, SD] (Oblak et al., 2022)	*P*-value (FDR-adjusted), significance level
Rationality	6.89 (98)	0.14 [0.43]	1.7 [2.21]	0.002, ***
Truth	5.42 (98)	1.17 [1.38]	1.97 [2.34]	0.356, NS
Fact	6.95 (93)	0.5 [1.19]	4.88 [5.83]	0.002, ***
Logic	6.11 (100)	0.17 [0.41]	0.65 [1.2]	0.057, NS
Objectivity	4.52 (100)	0.04 [0.15]	0.55 [0.78]	0.002, ***

NS, non-significant; ***p < 0.005.

Hernan’s focus on objectivity is reflected in his tendency for axiomatic language, phrasing his experience in terms of natural laws: “The present as well as the future can only exist upon the foundations of the past, meaning that each state of being is just a conclusion, a natural conclusion of the states of being that came before it.” Consider the following as well: “imagination and memory are going through the same pathways.”

### 4.4 Symptoms: phenomenology

The core aspect of Hernan’s psychopathology relates to his childhood maltreatment. His mother suffers from schizophrenia. Hernan reports his mother encouraging bullying among his siblings. At the beginning of his studies at university, upon coming out as homosexual to his family, his mother threw him out of the family’s apartment. This resulted in him living at the homeless shelter for nine months. Hernan is ambivalent about his period of displacement; he reports psychotic symptoms being diminished during this time, while experiencing homelessness as traumatizing.

Hernan experiences anxiety as the most detrimental for his everyday functioning (an assessment confirmed by his score on GAD-9; see Table 1). His anxiety is mostly related to his social life, and is severe enough that it commonly results in self-isolation. He quit his job as a journalist and started working from home because he did not have to interact with others. He relates his self-isolation to a decrease in depressive and anxious symptoms. Hernan developed maladaptive coping skills (e.g., almost ritual-like methodical approach for easing the discomfort of uncertainty). The symptoms as a whole and his upbringing contribute to his specific pattern of sensemaking that we call the crisis of objectivity (see below).

#### 4.4.1 Psychotic symptoms and anomalous experience of self and the world

Hernan’s EASE and EAWE scores are summarized in Table 3. Hernan scored 14 points on the EASE semi-structured interview. On EAWE, he scores 59 points, or 28 if we only account for schizophrenia-exclusive categories (captivation of attention by isolated details, loss of social common sense, alienated scrutinizing of others’ behavior, algorithmic approach to social understanding/interaction; intrusiveness of the gaze of another; tangential responding; disinclination for human society; adherence to abstract, intellectualistic, and/or autonomous rules; pervasive disbelief, skepticism, curiosity re the obvious/taken-for-granted; static quality, stillness, or morbid intellectualism; Sass et al., 2017). During the EASE interview, Hernan reported on the anomalous experience of self-awareness and presence. For example, he described the experience of diminished presence with a sense of barrier, a “filter” between himself and the external world:

**TABLE 3:** Scores on EAWE and EASE dimensions.

Scale	Score (w/o schizophrenia-exclusive categories)
EAWE	59 (28)
Space and objects	16 (11)
Time and events	5 (1)
Other persons	16 (8)
Language	2 (2)
Atmosphere	8 (4)
Existential orientation	3 (2)
EASE	14
Cognition and stream of consciousness	4
Self-awareness and presence	6
Bodily experience	4
Demarcation	0
Existential reorientation	0

For EAWE, scores are separated into total scores and those without symptoms present in other disorders than schizophrenia (in parentheses).

I will feel pushed back inside of my own mind and I would still receive the information from my senses, touch, taste, vision, etc. But I’ll feel it like muffled, like there was a filter between the information and me. I will still receive it [the information] but not feel like it was generally perceived by me.

He also described anomalous experience of his own embodiment in a sense of psycho-physical split, describing his body as a “meat suit”.

#### 4.4.2 “I used to be on fire, now only the ashes remain”: making sense of a history of mental disorder

A central aspect of Hernans’s self-narrative is that he is a person suffering from a mental disorder. He had been diagnosed with schizoaffective disorder and experienced at least one psychotic episode:

[O]ut of nowhere, my reflection in the mirror stopped, like, moving and talking. […] I couldn’t escape the thrall of the mirror. […] [T]he entity in the mirror just kept talking to me and was extremely nasty and telling me how I’m worthless and I deserve everything that happened to me and that I should kill myself.

Hernan regularly experiences simple visual pseudohallucinations, most typically appearing as anomalous textures, as well as imperative auditory hallucinations. Notably, he hears a voice saying the word “peel,” which prompts him to self-harm by scratching his skin. He also frequently experiences parasomnias, associated with falling asleep or waking up. The parasomniac hallucinations are veridical and emotionally congruent. Upon waking up, in the middle of the night, he often fights his husband, whom he mistakes for a hallucination:

It was a monster or a home invader that I was seeing. […] And biologically, the body is [] geared up to react to a threat. […] So, I find myself in the middle of what could be the most stressful situation any person can find himself themselves in, which is fighting for what I believe to be my survival, except that I find myself thrust in the middle of that fight, in the middle of that stress, without experiencing consciously all the steps leading up to that fight.

#### 4.4.3 Falling into the sunken place: symptoms of anxiety

Hernan himself refers to dissociation in social settings as “episodes”, and compares them to the Sunken Place from the movie *Get Out* (Peele, 2017). It is an exclusively negatively valenced experience that takes place in social settings and has a typical temporal dynamic: *rising phase, peak phase*, and *break.* During the rising phase, Hernan has insight into what is happening:

Stuff [is] pulling away from me and I can see and feel myself entering this state. […]I want to get out of here. I need to get out of here. It’s bad to be here. I’m afraid. I’m uncomfortable.

The gradual transition into an episode allowed him to develop protective behaviors in public settings (e.g., while he was still a journalist, he was able to read out prepared questions from a list). During the peak phase, Hernan experiences a detachment from his surroundings. This is associated with perceptual anomalies (e.g., tunnel vision, fading of color intensity, spatial distortions). While Hernan is sensorially connected to his environment, he feels as if his surroundings are no longer accessible to him. Hernan feels that lived time breaks down (e.g., he experiences a sense of eternity) and has to interfere with it in order for it to stop. While in the peak phase, Hernan feels as if verbal strategies (e.g., commanding himself to snap out of it) are not effective regulators. Hernan experiences decreased insight and the episodes are not amenable to conscious reflection, and he no longer comprehends the words that are being spoken^2^. Break occurs either through sensory deprivation or a change in the level of consciousness (e.g., sleep).

#### 4.4.4 “There is no spoon in the microwave”: obsessive compulsive symptoms

Finally, Hernan exhibits clear-cut signs of OCS. OCS occur when he is engaged in a potentially dangerous situation (e.g., leaving a spoon in a microwave). The presence of a negative outcome (e.g., being hit by a car, falling down the stairs) prompts him to start thinking about the danger. He then to mistrust his own cognition (e.g., he is unable to rely on his working memory, informing him of having looked both ways before crossing the road):

I put [the soup] in a bowl inside of the microwave. […] I close the door. And then I opened the door again […] It’s the feeling [that] the only thing that’s left is my memory of it being the way it was. And so I wonder. *Did I leave the spoon inside and reopen?* Check. *No spoon.* I close it. I reopen it. No spoon. […] It’s fear that something’s not right. […] I must have spent so much time of my life just opening and closing the stupid microwave.

Hernan is aware that his behavior is unreasonable, however, he is unable to stop himself: “I am […] rationally aware that this is not a normal fear to have−that is, there is no point that I’m being ridiculous and well, I’m being ridiculous.” Hernan experiences compulsions (e.g., making a small movement with his fingers above the bowl of soup in the microwave) as a form of irrationality. Yielding to them is a form of defeat for him.

### 4.5 Crisis of objectivity

The core of Hernan’s experience is what we term the *crisis of objectivity* (CoO). CoO is a pattern of sensemaking wherein Hernan mistrusts his own cognition. Hernan himself experiences CoO as the baseline aspect of consciousness: “In general, that’s an undercurrent. Not trusting myself is […] the catchphrase of my life at this point.” We can relate CoO to his trauma of growing up with a parent with schizophrenia: “My own mother is completely confused in her head. […] And I said I didn’t want to be like them, and to not be like them, I needed to be [pause] intellectually better.”

In his youth, Hernan’s only source of solace was the media. He notes that obtaining an education represented finding a better life away from his family. Striving for objectivity was also closely tied to his journalistic profession: “My job is literally to be as accurate as possible because other people rely on me to have access to objective reality.” Thus, for Hernan, objectivity is the paramount value:

Even if it’s something that requires a lot of time because like having to ponder and take apart the thread of a very difficult knot of, of threads that are stuck together, it can take months of, you know, regularly taking time just to myself, staring at the ceiling, laying down, doing nothing, just thinking for hours and hours and hours until I find an answer. Because an answer exists. There’s always an answer.

Having had problems with his mental health in general, but psychotic experience specifically, Hernan exists with the awareness that his judgments might be false. He distrusts himself not only when experiencing intrusive thoughts — what he calls the “call of the void”−but in every moment of reflection:

[T]here’s always the thought in the back of my head […] [t]here’s always a thought that my body is not just mine. Something could happen at any point […] [that] is a genuine threat to myself. […] [E]ach new experience not just reinforces this knowledge that my body is not trustworthy […] It is objectively true no matter how much you think things through.

While Hernan generally believes his senses, he has had experiences where this perceptual security was put into question. Thus, his baseline experience is that of the constant probability that his senses and cognition are false:

I have a better imagination than I do a memory. […] It’s being able to tell to myself: *I just checked [the microwave]*. *There is no, there is no spoon*. And I’m able […] to remember that I did [check it]. […] [T]he problem is that even though I have the ability to have a visual projection of the memory, it’s worthless because of the nature of the memory. The memory is not true. […] It will mean nothing because I know what the memory is and I know that every aspect of the memory is artificial.

Hernan is unable to trust his instincts. Rather, he has to reason about everything:

Because I hold myself to that standard that I don’t want my mind to become a barren wasteland no matter what genetic or what predisposition I have. […] I force myself to never, you know, go with my instinct, do what my mother would do.

CoO was apparent both in his reports on his daily life experience as well as his voluntary comments when performing the working memory task. Despite scoring in the 83rd percentile, Hernan continuously vocalized doubts about his task performance.

### 4.6 Ruminative introspection

Hernan engages in the process of *ruminative introspection* (RI). In response to a stressful event, Hernan reflects on this event in a highly ritualized fashion. His RI follows a specific set of steps:

(1) Hernan isolates himself and tries to minimize the presence of sensory stimuli, in particular, bodily sensations;
(2) RI is associated with a change in the level of consciousness:
(3) Hernan reflects on various events:
  (4.1) Hernan allows new ideas, related to the situation that he is reflecting on, to arise;
  (4.2) Once Hernan zeroes in on the topic, his rumination starts to deepen:

I try to force myself to empathize as if someone else was observing me and had all the correct information. […] What would they think? How would they judge me? If that person was not me, if that person did not have all of my biases, and all of my trauma and all of my problems.

Hernan relates subjective events to objective facts, so he is able to allocate responsibility in difficult social interactions:

[S]ince a lot of my socialization is done online, it’s been very practical for me, because I can literally go back into the text conversation and read the history of the conversations. […] I say: *did I do the right thing? Did the other person do the right thing?* […] One of the elements that led to our falling out [with best friend]was that I was doing [a gift] for him. I didn’t do [it]in time […] and he got really angry […] So, first I called back to memory, the events that occurred. Meaning every conversation, every interaction, when the topic was brought up by my best friend or me. […] When did I start working on it? How many hours of work did I get done? […] I was working two full-time jobs at the same time. And I just didn’t have the time. So, this is a fact. As objective a fact can be. […] [I]t can take weeks to go through every question that I want to ask myself. And go through all the facts and all the beliefs and all the biases and all the perceptions that are relevant to the topic.

Whenever Hernan is ruminating on specific social interactions, he is attempting to evaluate the statements that interlocutors had made according to formal logic:

[M]y filter is made out of knowledge, logical fallacies, about my own abilities. […] If I can say: *oh, this is, erm, appeal to authority.*

(5) Hernan reifies conclusions of RI (i.e., phrases it as a statement or a syllogism) and commits it to memory:

So what I do is I try to logically spell out for myself, Um, it’s like having a word document inside my head. I keep adding notes and I remember all the notes I’ve taken and I keep adding more and more and trying to put structure and logic in the way I think.

Hernan associates reaching a positive result of RI with positive feelings. Hernan noted that he might one day write a book of all of his realizations, specifically as a form of revenge against his mother.

### 4.7 Hernan’s personalized network model

**FIGURE 1:**
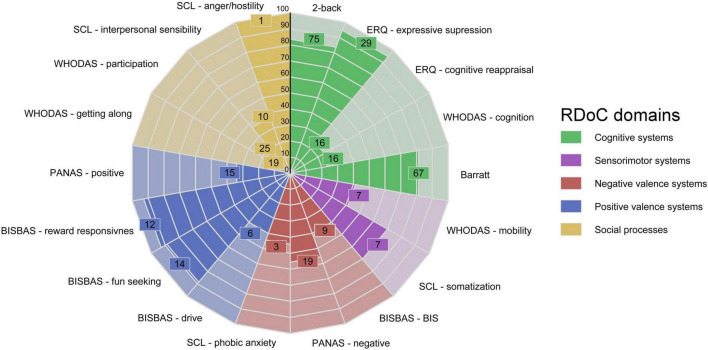
Hernan’s personalized network model. Areas in purple denote symptoms. Areas in blue denote patterns of sensemaking. Areas in red denote formative experiences. Areas in green denote biological factors. Areas in yellow denote social factors. Connections are made only between nodes where we can provide explicit data. Nodes are directed, wherein the starting node regulates the target node. +: upregulating node; –: downregulating node; OCS, Obsessive-compulsive symptoms; Anx, Anxiety; P&P, Psychotic symptoms and parasomnias; Fall in SP, Fall in Sunken Place; CoO, Crisis of Objectivity; SM, Sensemaking of himself as a person with mental disorder; RI, Ruminative introspection; ACE, Adverse Childhood Experience.

We observed stable relationships between different aspects of Hernan’s experience. Specifically, we analyzed which experiential categories *upregulate* others (i.e., make them more pronounced), and which *downregulate* them (make them less pronounced). Hernan’s personalized network model is outlined in Figure 1.

Hernan’s PNM is rhizomatic (as per our assumptions): all nodes are connected but no one node is connected to all others. When outlining Hernan’s PNM, it became apparent that CoO represents the central, maladaptive pattern of sensemaking, as it is present in the highest number of connections (*N* = 7). The mistrust into his own cognition was brought about by a) his traumatic experience of being a child of a patient with schizophrenia; and b) having himself suffered a psychotic experience in earnest. CoO is then reinforced by his parasomniac and anxious symptoms. The CoO forms the basis of his OCS, and is subsequently reinforced by what Hernan perceives as “yielding to irrationality” inherent in compulsions.

Interestingly, Hernan reports on CoO being beneficial when dealing with his anxiety:

I want to avoid the mess and the clutter [and] stupidity that I’ve seen in adults when I was a kid. […] And my whole life I’ve been trying to build myself structure, a mental structure, to rely on where my instinct might fail me.

Further, the process of RI downregulates both his feelings of anxiety and *Falling into the Sunken Place.* For an example of the former consider this:

When I am deep in this thinking, I am a lot less anxious. […] Just spending time by myself and thinking very hard about things, um, because the dark period was triggered by the outside events.

For an example of the latter, consider the following:

It dissipated after […] looking around myself and getting a sense of where I am […] [T]here are all the coping mechanisms that come with realizing something is in my head that we start with having to dissociate myself […] I also determine that everything I feel and everything I believe at the moment is fake. […] And so as soon as I can determine something is in my head, there is an entire process […] [I am] taking a moment to address one by one the actual feelings that I’m going through and taking long breaths, sitting down, looking around myself a lot to make sure that there really is nothing there.

## 5 Discussion

We presented a case study of Hernan, a patient who exhibits various symptoms precluding straightforward diagnosis and psychotherapeutic treatment. The goal of this paper was to see whether contemporary approaches in psychopathology (phenomenological psychopathology, PNMs, RDoC) can aid in diagnosis; to demonstrate how PNMs could be integrated into qualitative phenomenological research; and to illustrate a novel aspect of experience identified by PNM (*crisis of objectivity*) In the discussion, we will contextualize our findings within these three approaches, discussing Hernan’s condition as a disorder of the self, disorder of sensemaking, as well as how his condition fits within domains of functioning, described by RDoC.

### 5.1 The phenomenological perspective on self-disorders

The distinction between the minimal self (or ipseity) and narrative self is crucial in phenomenological psychopathology. The minimal self relates to personal experiences, while the narrative self encompasses one’s identity through stories and beliefs (Hutto, 2016). In psychotic spectrum disorders the minimal self is disrupted, evident in symptoms like impaired sense of agency and extracampine hallucinations (Chan and Rossor, 2002).

While schizoaffective disorder lacks robust evidence for anomalous self-experience, growing data suggest overlaps in phenomenology, biology, and genetics between schizophrenia and bipolar disorder, challenging their distinct diagnostic boundaries (Keshavan et al., 2011). Hernan’s EASE score was 14, compared to averages of 20.7 for schizophrenia and 6.3 for bipolar disorder as per Henriksen et al. (2021). Hernan exhibits disturbances of minimal self, which are not present equally in all relevant domains. This places Hernan on a milder end of the schizophreniform spectrum, which is consistent with his diagnosis of schizoaffective disorder (Henriksen et al., 2021).

Further, Hernan has difficulties trusting information that he appraises as being *subpersonal.* In the 20th century, mind sciences revealed that much of our psychology operates below conscious awareness. For example, in cognitive science, intuition relies on implicit learning of statistical patterns (Damassio, 1996). Neuroscience similarly reveals that the insula in the brain processes information from internal organs (Seth, 2013). While some of this leads to conscious awareness (interoception), only a fraction reaches the temporoparietal junction for conceptual processing of bodily schemas (Quesque and Brass, 2019). Thus, Hernan’s *Crisis of Objectivity* reflects a disturbance in what we could refer to as “cognitive” self, mistrusting subpersonal processes such as intuition. This echoes Cartesian doubt and a loss of perceptual safety:

If I’m carrying something, no matter the item, like just carrying groceries or, or a roll of toilet paper in my hand, I’m going to not trust myself to continue holding that item. Even if there are no consequences to it, I will still feel afraid and stressed at the thought of dropping it. […] The mere notion of me not trusting myself is a source of stress.

The pervasive sense of doubt has been identified as a central aspect of obsessive-compulsive psychopathology. Already in Pierre Janet’s description of OCS, doubt and feelings of uncertainty play a predominant role in understanding this disease (Pitman, 1987). These observations were confirmed by recent studies. Samuels et al. (2017) report on a large-scale survey that demonstrates pervasive doubting is associated with checking symptoms of OCS, as well as depression and anxiety. Chiang and Purdon (2023) report on semi-structured interviews that demonstrate OCS is associated with one’s doubting whether they performed certain tasks well, as well as more profound questioning of their own memory and perception (what we termed CoO).

From a phenomenological perspective, CoO may be linked to an underlying self-disorder. In the following quotation, we see how the dynamics of CoO is established. CoO could be also interpreted as an experience, leading into *lack of natural evidence* and subsequent *hyperreflexivity*, as it is described in self-disorders (Parnas and Sass, 2001) Furthermore, we could suspect that at least some of his rumination processes, described in the category of RI, seem to be of secondary nature as a consequence of underlying hyperreflexivity. Hyperreflexivity is one of the fundamental components of ipseity disturbances. Consequently, aspects of oneself are experienced as a kind of external object (Sass and Parnas, 2003).

Hernan presents with OCS as well as psychotic symptoms. The co-occurrence of both pathologies was recognized as early as the first descriptions of schizophrenia (Bleuler, 1911). Stengel (1945) also speculated that in patients with schizophrenia the psychotic reaction was kept under control with the aid of obsessional symptoms. From the phenomenological perspective, the feature that is usually thought to be crucial for differentiating an obsession from a delusion is insight (De Haan et al., 2013). However, individuals with OCS are prone to confusion between reality and possibility; they tend to mistake hypothetical possibilities for real probabilities, which has been termed inferential confusion (Aardema et al., 2005). The crucial aspect of the latter is distrust of the senses, also seen in our patient. Some authors state that the compulsive adherence to the doubt in the obsessive patient could be an equivalent to the absolute certainty in a delusion of the psychotic patient (Dalle Luche and Iazzetta, 2008) and it has even been suggested that OCD could be better characterized as a belief disorder (Ahern et al., 2019).

### 5.2 RDoC perspective

The problem of symptom heterogeneity can be tackled through the RDoC framework. Hernan displays an idiosyncratic pattern of maladaptive sensemaking. The question is whether this maladaptive pattern of sensemaking constitutes a cognitive dysfunction that could be attributed to psychosis spectrum disorder (e.g., disorganization symptoms). Schizophrenia spectrum disorders present varied symptoms, notably executive function deficits (Torrent et al., 2007; Gotra et al., 2020), affecting processing speed, memory, attention and reasoning. These deficits persist over time and are not attributable to antipsychotic treatments (Green et al., 2019).

Hallucinations, in particular auditory hallucinations, are considered to be disruptions in multiple domains in the RDoC framework. They implicate the cognitive domain at the level of language, perception, declarative memory, and cognitive control. On the level of social domain, they are related to affiliation (RdoC construct that refers to positive interactions with others) and perception and understanding of self (agency). Finally, in the negative valence systems, hallucinations are related to both sustained and acute threat (Ford et al., 2014). Within perception (a cognitive systems domain), dysfunctions of sensory integration are well-document in psychosis spectrum disorders. A recent RDoC-informed study has demonstrated that visual integration dysfunction is a symptom that is general across psychosis spectrum disorders and not characteristic only of schizophrenia (Grove et al., 2018).

In Hernan, we observed some symptoms that could be linked to motor systems disorders. During interactions, he exhibits twitches, self-harming behavior (scratching) and often interrupts the interviewers. Sensorimotor dysfunctions may serve as a biomarker for psychosis spectrum disorders (Mittal et al., 2017; Hirjak et al., 2018). Patients exhibit varied movement disorders, including velocity scaling, stereotypies, catatonic immobility, and perseveration. Smooth movement execution and motor plan updating are disrupted, manifesting as poor postural control, motor learning issues, and eye-blink conditioning. Schizophrenia shows psychomotor slowing, affecting emotional and motor regulation. This can result in varied movement patterns and catatonia.

RDoC-based research highlights impaired facial recognition, especially fear and anger detection, in psychosis spectrum disorders like schizophrenia (Gur and Gur, 2016; Gur et al., 2017). Emotion recognition, including gaze perception, is a common transdiagnostic biomarker (Tso et al., 2020). Hernan exhibits issues with eye contact and scores on social cognition scales like WHODAS and SCL indicate challenges in this area.

Discrimination (on the basis of gender, race, creed, etc.) is a risk factor for psychopathology during development, perhaps by putting additional demands on implicit emotional regulation (Vargas and Mittal, 2021). Being homosexual and an immigrant may have played a modifying role in Hernan’s psychopathology. Psychotic spectrum disorders show impaired performance in a variety of cognitive control tasks (Sabharwal et al., 2016; Smucny et al., 2018). Finally, rumination, linked to worse psychiatric prognosis and severity, correlates with socioeconomic status (Silveira et al., 2020).

Bayer et al. (2023) propose language production as a reliable measure of thought disorder, linking positive disorder to semantic coherence and negative disorder to syntactic complexity. Psychosis spectrum disorders impact speech and social function but not mood disorders (Cohen et al., 2012). Hernan shows fluent speech with complex ideas but displays positive thought disorder, acknowledging his “meandering” thinking style.

Within the RDoC framework, anxiety aligns with danger detection in the negative valence domain. OCS is viewed as a maladaptive system hyperactivity evolved for detecting danger (Woody et al., 2019). Figee et al. (2016) connect OCS to behavioral addiction and maladaptive reward systems. Common neurobiological mechanisms exist between OCS and anxiety (Gillan et al., 2017). Compulsions are also linked to impulsivity, a component of cognitive control (Moreno-Montoya et al., 2022).

### 5.3 Methodological considerations

The present paper represents an attempt to put the idea of PNMs, originally introduced in De Haan (2020), into practice. In this study, we employed PNMs as a conceptual framework for qualitative analysis. As such, our approach amounts to a somewhat narrow use of PNMs (which could also be employed within a quantitative framework by using dynamical systems or graph theory). Nonetheless, we found using PNMs as a guiding principle for qualitative analysis useful. In doing so, we made several methodological observations, which may be of use to other researchers.

Firstly, using PNMs as a qualitative framework necessitates that both the nodes and connections be grounded in the data. In our experience, this requires a somewhat larger amount of data collection than is typical for qualitative studies. We were only able to ground all the relevant experiential categories after 14 interview sessions (from our own experience with qualitative phenomenology, on average, 11 participants−assuming one interview per participant−are sufficient to exhaust all the relevant qualitative material). Even after 14 interview sessions, two connections were only observed once, and were thus omitted from the final presentation of the data.

Secondly, it became apparent early in the study that grounding all the connections in the qualitative material would be prohibitively complex without adopting the simplifying assumption of them referring to up- and downregulation. It is likely that establishing more complex connections would require either a) further data collection; or b) adopt additional data collection techniques.

**FIGURE 2:**
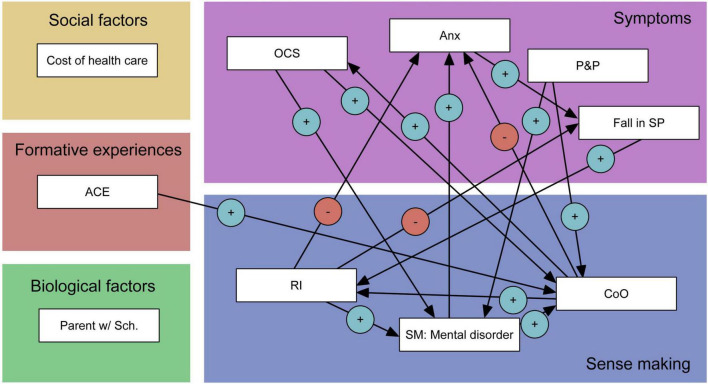
Hernan’s scores on the mini RDoC battery. Green: cognitive systems; red: negative valence systems; blue: positive valence systems; yellow: social processes. Areas with saturated colors denote Hernan’s percentile rank on a given construct. Percentile ranks are represented by colored areas. Hernan’s absolute scores on given constructs are marked with white numerals. Higher values represent greater presence of the psychological function in question; thus scores on SCL, WHODAS, and Barratt were inverted to reflect this principle.

Thirdly, as evident in Figure 2, our PNMs primarily consist of data collected at the same level of description: lived experience. Establishing connections with other domains (social and biological) may require non-qualitative data collection strategies (e.g., experimental research design).

An open question remains how we might translate the collected qualitative material into quantifiable data.

## 6 Limitations

In the present study, we sought to further develop the methodological framework of PNMs, originally proposed by De Haan (2020) and Larsen et al. (2022a,b). In this study, we used the data from a patient, chosen due to the complexity of his clinical presentation and his willingness to participate in the study in the long-term. Our approach has two critical limitations. Firstly, while we collected quantitative measures (i.e., questionnaires) and objective data (i.e., formal linguistic analysis and cognitive task measures) in addition to qualitative phenomenological reports, these are not used in the PNMs. Rather, they serve as objective validations of patterns observed in the PNMs. Future work on PNMs should focus on integrating quantitative measurements as well as rely more heavily on graph theory in constructing them. Second, Hernan was originally recruited for a study on online psychotherapy. As such, we were unable to perform clinical tests (e.g., bloodwork and neuroimaging). Additionally, as is well-known, clinical interviews that are not conducted in person are of lower quality and validity as they lack intersubjective attunement and implicit countertransferal dynamics (Jansson and Nordgaard, 2016).

## 7 Concluding remarks

Symptom heterogeneity constitutes a challenging problem in psychiatry, both in terms of how best to research specific psychiatric disorders and treat them in a clinical setting. Recently, several frameworks have been proposed to tackle the problem of psychiatric comorbidities. We presented a case study where several approaches were used in order to identify the core of his pathology. We employed an in-depth mixed methods approach to describe his psychopathology. We identified one experiential category−the *crisis of objectivity*−as the core psychopathological theme of his lifeworld. CoO refers to his persistent mistrust towards any information that he obtains that he appraises as originating in his subjectivity. We can developmentally trace CoO to his adverse childhood experience, as well as him experiencing a psychotic episode in earnest. Hernan developed various maladaptive coping mechanisms in order to compensate for his psychotic symptoms. Interestingly, we found correspondence between his subjective reports and other sources of data. Hernan exhibits difficulties in multiple RDoC domains. While we can say that social, sensorimotor, positive valence, and negative valence systems dysfunctions are likely associated with primary deficit (originating in his adverse childhood experience), his cognitive symptoms may be tied to his maladaptive coping mechanisms. Our multi-method approach demonstrates how multiple sources of data may converge onto novel understanding of symptom clusters by identifying their common core. It would be of interest to see whether such an approach can be further used for personalized treatment of psychiatric disorders and whether it can be scaled up to include enough patients to be representative of relevant populations.

## Data availability statement

The datasets presented in this study can be found in online repositories, https://osf.io/dj8pt/. The names of the repository/repositories and accession number(s) can be found in the article/supplementary material.

## Ethics statement

The studies involving humans were approved by the University Psychiatric Clinic Ljubljana Review Board. The studies were conducted in accordance with the local legislation and institutional requirements. The participants provided their written informed consent to participate in this study. Written informed consent was obtained from the individual(s) for the publication of any potentially identifiable images or data included in this article.

## Author contributions

AO: Conceptualization, Data curation, Formal analysis, Investigation, Methodology, Project administration, Software, Supervision, Visualization, Writing−original draft, Writing−review and editing. MK: Conceptualization, Writing−review and editing. KHG: Investigation, Writing−original draft, Writing−review and editing. AH: Investigation, Writing−original draft, Writing−review and editing. UB: Supervision, Writing−review and editing. BS̆: Supervision, Writing−review and editing. JB: Conceptualization, Funding acquisition, Supervision, Writing−original draft, Writing−review and editing.

## Footnotes

1 To ensure Hernan’s anonymity, the transcripts will only be made available to researchers upon reasonable request.

2 Due to this, Falling Into the Sunken Place, as an experiential category, should be understood critically. We constructed it analytically, and not as a phenomenon directly reported by Hernan.
